# AMPK Activation Prevents and Reverses Drug-Induced Mitochondrial and Hepatocyte Injury by Promoting Mitochondrial Fusion and Function

**DOI:** 10.1371/journal.pone.0165638

**Published:** 2016-10-28

**Authors:** Sun Woo Sophie Kang, Ghada Haydar, Caitlin Taniane, Geoffrey Farrell, Irwin M. Arias, Jennifer Lippincott-Schwartz, Dong Fu

**Affiliations:** 1 Faculty of Pharmacy, The University of Sydney, Sydney, NSW, Australia; 2 Liver Research Group, Australian National University Medical School, Canberra, Australia; 3 National Institute of Child Health and Human Development, National Institutes of Health, Bethesda, Maryland, United States of America; 4 Janelia Research Campus, Howard Hughes Medical Institute, Ashburn, Virginia, United States of America; Heinrich-Heine-Universität Düsseldorf, GERMANY

## Abstract

Mitochondrial damage is the major factor underlying drug-induced liver disease but whether conditions that thwart mitochondrial injury can prevent or reverse drug-induced liver damage is unclear. A key molecule regulating mitochondria quality control is AMP activated kinase (AMPK). When activated, AMPK causes mitochondria to elongate/fuse and proliferate, with mitochondria now producing more ATP and less reactive oxygen species. Autophagy is also triggered, a process capable of removing damaged/defective mitochondria. To explore whether AMPK activation could potentially prevent or reverse the effects of drug-induced mitochondrial and hepatocellular damage, we added an AMPK activator to collagen sandwich cultures of rat and human hepatocytes exposed to the hepatotoxic drugs, acetaminophen or diclofenac. In the absence of AMPK activation, the drugs caused hepatocytes to lose polarized morphology and have significantly decreased ATP levels and viability. At the subcellular level, mitochondria underwent fragmentation and had decreased membrane potential due to decreased expression of the mitochondrial fusion proteins Mfn1, 2 and/or Opa1. Adding AICAR, a specific AMPK activator, at the time of drug exposure prevented and reversed these effects. The mitochondria became highly fused and ATP production increased, and hepatocytes maintained polarized morphology. In exploring the mechanism responsible for this preventive and reversal effect, we found that AMPK activation prevented drug-mediated decreases in Mfn1, 2 and Opa1. AMPK activation also stimulated autophagy/mitophagy, most significantly in acetaminophen-treated cells. These results suggest that activation of AMPK prevents/reverses drug-induced mitochondrial and hepatocellular damage through regulation of mitochondrial fusion and autophagy, making it a potentially valuable approach for treatment of drug-induced liver injury.

## Introduction

Drug-induced liver injury (DILI) underlies 50% of all cases of acute liver failure, and is caused by many structurally and functionally unrelated drugs. DILI has limited treatment options and represents the main reason for withdrawal of new agents during drug development or after approval [1–3]. Although DILI’s underlying cellular basis is poorly understood, the key cellular player is the mitochondria [4–6]. Mitochondrial injury is widespread in DILI, with drugs and their toxic metabolites causing striking changes in mitochondria behaviour and function. These changes have been used to screen drug candidates for hepatotoxicity [7] and include the appearance of mitochondrial fragments, reduced mitochondrial membrane potential and release of pro-apoptotic proteins into the cytosol [8–11]. Given that these effects on mitochondria can prompt apoptosis and/or tissue necrosis, better understanding of the relationship between mitochondria injury and DILI is warranted. Indeed, hepatocyte survival and therapeutic outcome in DILI may benefit from treatments that minimize mitochondrial damage and/or enhance mitochondrial function.

A mitochondrial quality control system maintains healthy mitochondria within cells. This system is comprised of two arms: one regulates mitochondrial fusion/fission dynamics and the other regulates mitochondrial turnover by autophagy (mitophagy) [12, 13]. Fusion/fission requires the outer membrane fusion proteins, mitofusion protein 1 and 2 (Mfn1, 2), the inner membrane fusion protein optic atrophy type 1 (Opa1) and the fission protein dynamin-related protein 1 (Drp1), which requires phosphorylation for activation [14]. Mitophagy requires the core autophagy machinery and Pink/Parkin regulators for degradation of mitochondria [15]. When a mitochondrion is under minor stress, fusion with a healthy mitochondrion is thought to complement the damaged components and maintain its function. When mitochondria experience additional stress and are more substantially damaged, fission is activated and separates healthy and damaged mitochondria. The damaged mitochondria are degraded by mitophagy, leaving healthy ‘daughter’ mitochondria free to fuse and function normally. Meanwhile, recycled biomaterials from mitophagy can be used to synthesize new mitochondria which subsequently fuse [12]. Through such ‘quality control’, mitochondria minimize or eliminate damage from intra- and extracellular stress.

A key factor in maintaining and producing healthy mitochondria is AMP activated kinase (AMPK), the cell’s major energy sensor [16, 17]. Activated by phosphorylating Thr-172 of the α-subunit, AMPK promotes mitochondrial biogenesis and function by increasing the transcriptional activity of peroxisome proliferator-activated receptor-γ co-activator 1α (PGC-1α), which enhances transcription of nuclear-encoded mitochondrial genes [18–20]. Activation of AMPK also switches on autophagy which removes damaged mitochondria from cells [21].

Given AMPK’s role in mitochondria biogenesis and autophagy, we studied whether AMPK activation could prevent and/or reverse drug-induced hepatocyte injury. We used rat or human hepatocytes cultured in a collagen sandwich. The collagen sandwich culture of hepatocytes forms a multicellular canalicular network as seen in vivo, and maintains this polarized morphology as well as activities of enzymes and transporters for ~2–3 weeks whereas other culture conditions result in rapid deterioration of primary hepatocytes [22, 23]. The commonly used analgesics, acetaminophen and diclofenac, were used to induce hepatocyte injury in the collagen sandwich cultures. Acetaminophen (APAP) is the leading cause of DILI worldwide [9, 24, 25], and causes hepatocyte injury via its metabolite NAPQI (*N*-acetyl-*p*-benzoquinone imine). Diclofenac (Diclo) causes idiosyncratic DILI by unknown mechanisms. After demonstrating the damaging effects of these drugs in hepatocyte cultures, we tested whether co-treatment with the specific AMPK activator AICAR [26, 27] could alter the outcome. Strikingly, the mitochondria becoming fused and active for ATP production, and the cells maintained polarized morphology and survived. AMPK activation prevented drug-mediated decreases in mito-fusion proteins Mfn1, 2 and Opa1 and stimulated autophagy. Moreover, AMPK activation also reversed the drug-induced hepatocyte injury before it reaches irreversible damage. Thus, activation of AMPK may be a valuable approach for treatment of drug-induced liver injury.

## Materials and Methods

### Chemicals and reagents

AICAR (5-Aminoimidazole-4-carboxamide ribonucleotide), rapamycin, diclofenac, carbonyl cyanide 4-(trifluoromethoxy) phenylhydrazone (FCCP) and collagenase were purchased from Sigma Aldrich. Acetaminophen was purchased from Bronson & Jacobs (Villawood, Australia). MitoTracker green, tetramethylrhodamine ethyl ester (TMRE), Hoechst 33342 and ATP determination kit were purchased from Invitrogen. Anti-Mitofusion-1 and anti-Tom20 antibodies were from Santa Cruz Biotechnology. Anti-Mitofusin-2, anti-AMPK alpha, anti-Phospho-AMPK alpha (Thr172), anti-ACC (Acetyl-CoA Carboxylase), anti-phospho-ACC (ser79), anti-Drp1, anti-phospho-Drp1 (ser616), anti-LC3 and anti-Parkin antibodies were from Cell Signaling Technology. Anti-OPA1 antibody was from BD Transduction Lab. Mouse anti-ABCB1 (C219) antibody was from Covance Research and rabbit anti-occludin antibody was purchased from Invitrogen. Secondary HRP-conjugated goat anti-rabbit and anti-mouse IgG were bought from Jackson ImmunoResearch. Anti-rabbit Alexa fluro 488 and anti-mouse CY3 antibodies were from Invitrogen. Clarity^TM^ ECL western blotting substrate was from Bio-Rad.

### Isolation of rat hepatocytes and collagen sandwich culture of human or rat hepatocytes

Sprague Dawley rats were obtained from Animal Resources Centre, Perth, Australia. As described in previous studies [28], male 250g Sprague Dawley rats were anesthetized. The liver was perfused with buffer and collagenase A solution (Sigma). Rats died under the general anesthesia during the non-survival surgery. Rat hepatocytes were isolated after the liver perfusion [28]. The protocol was approved by Animal Ethics Committee of the University of Sydney (protocol number L24/10-2012/3/5864). Plateable human hepatocytes were obtained from Invitrogen, and certified for metabolic enzymes activities (HMCPMS, Lot HU1455 and HU1539, donors information http://tools.lifetechnologies.com/content/sfs/COAPDFs/2013/HU1455_HMCPMS.pdf and http://tools.lifetechnologies.com/content/sfs/COAPDFs/2013/HU1539_HMCPMS.pdf). Rat or human hepatocytes were seeded at a density of 2×10^5^ on a 14mm microwell of 35mm glass-bottom dishes (MatTek, Ashland, MA) that were pre-coated with type 1 collagen. After overnight culture in 37°C incubator in 5% CO_2_, collagen was overlaid on top the hepatocytes which were further cultured for 5 days and were ready for treatments.

### Cell viability assay

LIVE/DEAD viability/cytotoxicity Kit (Invitrogen) was used to exam cell viability. Following recommendation from the manufacture, working solution (2μM calcein AM and 4μM EthD-1 in PBS) was added to the collagen sandwich culture of hepatocytes which was then incubated for 15 minutes in 37°C incubator in 5% CO_2_. After quick washing with PBS, cells were immediately imaged using Olympus FluoView FV1000 confocal microscope with heated-stage insert and a 40X objective. At least three independent experiments were conducted. Images were taken from multiple areas which were randomly selected within the dishes in each treatment. ImageJ (NIH, Bethesda, MD) was used to analyse the images blind to the treatments. Live cells were stained as green and only nuclei from dead cells were stained as red. Numbers of dead and live cells were counted and viabilities were calculated, and ~1000–2200 cells were counted for each treatment. Percentages of viability were obtained by normalizing to the control.

### Immunofluorescence assay and hepatocyte polarization morphology

Hepatocytes were fixed in 4% paraformaldehyde in PBS for 15 minutes followed by incubation with methanol for 5 minutes at -20°C, blocked and permeabilized with blocking buffer (1% BSA and 0.5% Triton X-100 in PBS) for an hour, and incubated with primary antibodies overnight at 4°C. Antibodies for apical marker ABCB1 and tight junctional marker occludin were used for morphological detection of hepatocytes polarization. After washing with PBS, cells were incubated with secondary antibodies for 90 minutes, followed by washing in PBS. Nuclei were stained by incubating with Hoechst 33342 solution. Confocal images were taken using Olympus FluoView FV1000 confocal microscope with a 60x objective. As previously described [28], dishes for the treatments were blinded and at least three areas were randomly imaged. The length of canaliculi within the projection images was measured using ImageJ. Canaliculi length was obtained by summing overall canalicular length and dividing by total cell number to obtain canalicular length per cell. Percentages of canalicular length were obtained by normalizing to the control. Mean ± s.d. was calculated from 3–4 individual experiments.

### ATP determination

Cellular ATP level was determined using an ATP bioluminescence assay kit (Invitrogen). After 24 hour treatment, cells were harvested and lysed. 10μl of the supernatant or standard ATP solution with 90μl of reaction buffer were added per well in a white 96 well assay plate for luminescence reading on BMG POLARstar Galaxy to determine ATP. Percentages of ATP were obtained by normalizing to the control. Mean ± s.d. was calculated from 3–4 individual experiments.

### Mitochondrial membrane potential

As described in previous studies [29, 30], to examine mitochondrial membrane potential, hepatocytes were incubated with 250nM of MitoTracker Green in culture medium for 15 min and then further incubated with 500nM tetramethylrhodamine ethylamine (TMRE) for 15 min. After washing cells with PBS three times, images were obtained with Olympus FluoView FV1000 confocal microscope equipped with a heating system. The ratios of fluorescent density of 543-nm to 488-nm channels were calculated using ImageJ. Percentages of mitochondrial potential were obtained by normalizing to the control. Mean ± s.d. was calculated from 3–4 individual experiments.

### Mitochondrial morphology

After staining with MitoTracker Green and TMRE or immunofluorescence using anti-Tom20 primary antibody, images were taken using Leica SP5II confocal microscope with a 100X objective. Merged projection images were used to study mitochondrial morphology. Mitochondria morphology was scored based on the shape and ratio of length to width, and scoring was conducted blind to treatments. Mitochondria were first identified as ‘round’, ‘tubular’ and ‘interconnected’. Hepatocytes had interconnected mitochondria network were defined as ‘fused’. Hepatocytes had dominantly round spherical mitochondria were defined as ‘fragmented’. For hepatocytes only containing mixture of ‘round’ and ‘tubular’ mitochondria, the width and the length of a tubular mitochondrion were measured using ImageJ and the ratio of length to width was calculated. A mitochondrion was defined as ‘long tubular’ if the ratio was more than 10. Hepatocytes containing ≥5 long tubular mitochondria were counted as cells with fused mitochondria. Percentages of cells with fused mitochondria were calculated.

### Western blot

After various treatments, hepatocytes were lysed and total proteins were extracted. SDS-PAGE gel was run. After transfer, the PVDF membrane was incubated with primary antibody followed by incubation with secondary antibody and final developing using ECL solution. Details were described as previously [29, 31]. Percentages of density were obtained by normalizing to the control. Mean ± s.d. was calculated from 3–4 individual experiments.

### Statistical analysis

Two-tailed student’s T-test was used to analyze statistical significance.

## Results

### AMPK activation prevents acetaminophen and diclofenac-induced hepatocyte injury and depolarization

**Fig 1:**
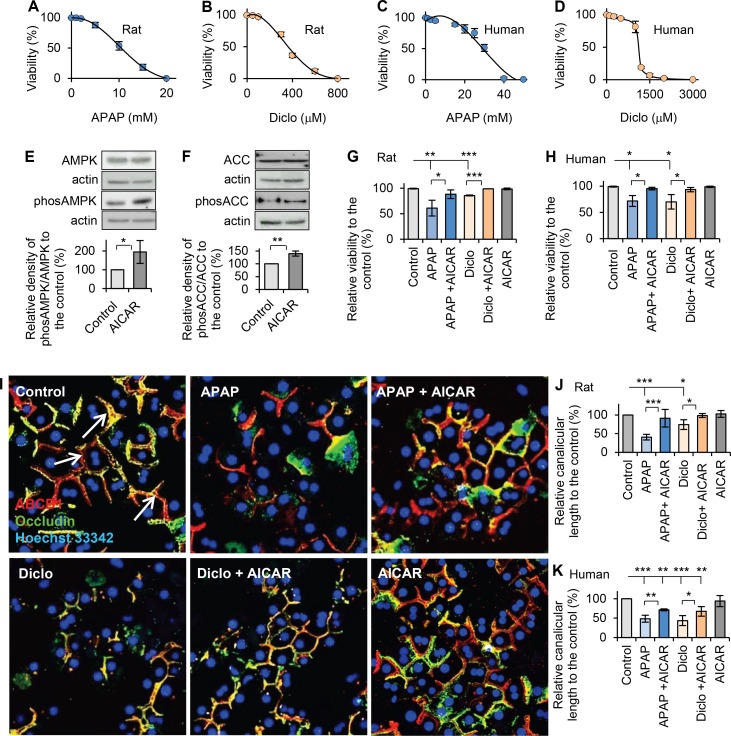
Activation of AMPK prevented drug-induced hepatocellular injury and hepatocyte depolarization. Using LIVE/DEAD viability/cytotoxicity Kit (Invitrogen) and confocal microscope images, hepatocyte viabilities were analysed (see details in Methods). After hepatocytes were treated with various concentrations of acetaminophen (APAP) or diclofenac (Diclo), dose-viability response curves were obtained for (**A**) acetaminophen (1–20mM, 24hr) in rat hepatocytes, (**B**) diclofenac (50–800μM, 24hr) in rat hepatocytes, (**C**) acetaminophen (1–50mM, 24hr) in human hepatocytes, and (**D**) diclofenac (100–3000μM, 24hr) in human hepatocytes. (**E**) Western blot was used to detect the expressions of AMPK, phos-AMPK (Thr172), ACC and phos-ACC (Ser79). Quantitative analyses were conducted by calculating the relative densities compared to respective controls. 200μM AICAR significantly activated AMPK measured by the ratio of phos-AMPK (Thr172) to total AMPK (195% of that in the control), and (**F**) AICAR significantly increased the ratio of phos-ACC (ser79) to total ACC (140% of that in the control). (**G**) Rat hepatocytes were treated with acetaminophen (10mM, 24h) or diclofenac (250μM, 24h) in the presence or the absence of AICAR (200μM). Viabilities were measured and percentages of viability relative to the control were calculated. Both drugs significantly decreased viability, and AICAR prevented drug-induced decrease in viability (relative viabilities were: 61% in APAP; 88% in APAP + AICAR; 86% in Diclo, 99% in Diclo +AICAR and 99% in AICAR). (**H**) Human hepatocytes were treated with acetaminophen (25mM, 24h) or diclofenac (1000μM, 24h) in the presence or the absence of AICAR (200μM). Addition of AICAR prevented drug-induced decrease in viability (relative viabilities were: 72% in APAP; 95% in APAP + AICAR; 70% in Diclo, 93% in Diclo +AICAR and 99% in AICAR). (**I**) Immunofluorescence and confocal microscope were used to examine polarized morphology after various treatments in hepatocytes (see details in Methods). Representative images showed that acetaminophen and diclofenac depolarized hepatocytes resulting in less branched canalicular network in cells. AICAR prevented drug-induced depolarization in rat hepatocytes treated with acetaminophen or diclofenac. The white arrows indicate the representative canalicular network, which is the orange-yellow tubular structure within the hepatocytes. (**J**) Quantitative analyses of polarization were performed by measuring the canalicular lengths. Percentages of canalicular lengths relative to the control were calculated. Both drugs reduced the canalicular length. AICAR prevented the decrease in canalicular length in rat hepatocytes treated with acetaminophen (10mM, 24h) or diclofenac (250μM, 24h) (relative canalicular lengths were: 41% in APAP; 92% in APAP + AICAR; 74% in Diclo; 98% in Diclo + AICAR and 103% in AICAR). (**K**) AICAR prevented the decrease in canalicular length in human hepatocytes treated with acetaminophen (25mM, 24h) or diclofenac (1000μM, 24h) (relative canalicular lengths were: 48% in APAP; 71% in APAP + AICAR; 44% in Diclo; 68% in Diclo + AICAR and 94% in AICAR) (* p<0.05, ** p<0.01 and *** p<0.001).

To select the drug concentrations that cause intermediate (moderate but significant) hepatocyte damage, dose-response curves for cell death were obtained by measuring the viabilities of hepatocytes treated with different concentrations of acetaminophen or diclofenac for 24h. The LC_50_ in rat hepatocytes were ~11mM for acetaminophen and ~360μM for diclofenac; all rat hepatocytes were killed after exposure to ≥18mM acetaminophen or ≥600μM of diclofenac (Fig 1A and 1B). Therefore, we selected 10mM acetaminophen and 250μM diclofenac, which caused intermediate hepatocellular injury (viabilities: ~65% and ~78%, respectively), for subsequent studies in rat hepatocytes. In human hepatocytes, the LC_50_ were ~30mM for acetaminophen and ~1100μM for diclofenac (Fig 1C and 1D), which were higher than those in rat hepatocytes. Thus, 25mM acetaminophen and 1000μM diclofenac, which caused intermediate hepatocellular injury (viabilities: ~73% and ~80%, respectively), were used for subsequent studies in human hepatocytes. The LC_50_ values of acetaminophen and diclofenac were similar to those in previous reports [32, 33]. The clinical toxic serum concentration of acetaminophen is slightly lower than that obtained in our cultured human hepatocytes (~3mM vs. ~5–10mM) [34–36], which may result from added mechanisms, such as formation of reactive metabolites or possible immune mechanisms [35, 37], which increase the drug’s damaging effects in the whole organism.

To investigate whether AMPK activation prevents drug-induced hepatocellular injury, cell viability was measured after hepatocytes were co-incubated with 200μM AICAR, a specific activator of AMPK, and acetaminophen or diclofenac for 24hrs. Western blot results confirmed that 200μM AICAR significantly activated AMPK, measured by the ratio of phos-AMPK (Thr172) to total AMPK, to 195% of that in the control (Fig 1E). AICAR also significantly increased AMPK substrate phos-ACC (Ser79) level, measured by ratio of phos-ACC (Ser79) to total ACC, to 140% of that in the control (Fig 1F). Notably, addition of AICAR significantly improved the viability of rat and human hepatocytes treated with acetaminophen or diclofenac, and the viability increased to 88% and 99% in rat hepatocytes and 95% and 93% in human hepatocytes treated with acetaminophen and diclofenac, respectively (Fig 1G and 1H). These results suggest that AMPK activation prevented drug-induced hepatocyte injury.

Hepatocyte polarized morphology is important for hepatocytes to survive under stress. Thus, the polarization status was also investigated to indicate hepatocyte injury. Hepatocytes have apical and basolateral membrane domains. Tight junction proteins, such as occludin, claudin and ZO-1, seal the canalicular lumen resulting in separation of apical and basolateral membrane domains. The apical domains from adjacent hepatocytes form a tubular bile canaliculus which connects and develops a canalicular network [38]. Both acetaminophen and diclofenac treatments disrupted the polarized canalicular network in hepatocytes as examined by immunofluorescence and confocal microscopy. Addition of AICAR in hepatocytes treated with acetaminophen or diclofenac prevented depolarization and maintained the branched canalicular network (Fig 1I). Furthermore, canalicular length, a measure of hepatocyte polarization [28], was significantly reduced in drug treated hepatocytes. In hepatocytes treated with acetaminophen or diclofenac, the canalicular lengths decreased to 41% and 74% of that in the control rat hepatocytes, respectively, and 48% and 44% of that in the control human hepatocytes, respectively (Fig 1J and 1K). However, canalicular lengths significantly improved in both rat and human hepatocytes treated with acetaminophen or diclofenac in the presence of AICAR. Addition of AICAR increased canalicular lengths to 92% or 99% of that in control rat hepatocytes treated with acetaminophen or diclofenac, respectively; and 71% or 68% control in human hepatocytes treated with acetaminophen or diclofenac, respectively (Fig 1I, 1J and 1K). Thus, activation of AMPK by AICAR prevented hepatotoxic drug-induced depolarization and restored hepatocyte survival. This is consistent with AMPK’s key role in the formation and maintenance of hepatocyte polarization [28].

### AMPK activation protects hepatocytes against acetaminophen and diclofenac-induced mitochondrial dysfunction and fragmentation

**Fig 2:**
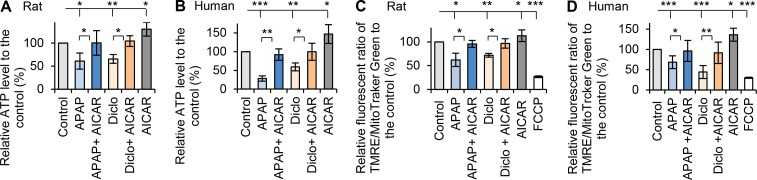
Activation of AMPK prevented drugs-induced mitochondrial dysfunction. Rat or human hepatocytes were treated with acetaminophen (APAP) or diclofenac (Diclo) in the presence or the absence of AMPK activator AICAR (200μM). Cellular ATP levels were measured using ATP determination kit (Invitrogen). Percentages relative to respective controls were calculated. (**A**) AICAR prevented the decrease in cellular ATP in rat hepatocytes treated with acetaminophen (10mM, 24h) or diclofenac (250μM, 24h). Treatment with AICAR alone significantly increased ATP level. The relative ATP were 61% in APAP; 100% in APAP + AICAR; 65% in Diclo; 104% in Diclo + AICAR and 130% in AICAR, and (**B**) AICAR prevented the decrease in ATP in human hepatocytes treated with acetaminophen (25mM, 24h) or diclofenac (1000μM, 24h). The relative ATP levels were 28% in APAP; 92% in APAP + AICAR; 59% in Diclo; 100% in Diclo + AICAR and 147% in AICAR. Treatment with AICAR alone significantly increased ATP level. (**C**) Mitochondrial potentials were measured and quantitatively analysed using Image J (see details in Methods). Percentages relative to respective controls were calculated. AICAR prevented the decrease in mitochondrial potential in rat hepatocytes treated with acetaminophen (10mM, 24h) or diclofenac (250μM, 24h). Treatment with AICAR alone significantly increased mitochondrial potential. FCCP treatment was used as negative control. The relative mitochondrial potentials were 62% in APAP; 96% in APAP + AICAR; 72% in Diclo; 97% in Diclo + AICAR; 113% in AICAR and 27% in FCCP. (**D**) AICAR also prevented the decreases of mitochondrial potential in human hepatocytes treated with acetaminophen (25mM, 24h) or diclofenac (1000μM, 24h). The relative mitochondrial potentials were 68% in APAP; 96% in APAP + AICAR; 44% in Diclo; 91% in Diclo + AICAR; 136% in AICAR and 30% in FCCP (* p<0.05, ** p<0.01 and *** p<0.001).

Previous studies suggested that both acetaminophen and diclofenac reduce mitochondrial membrane potential [39–41] and oxidative stress [42]. To examine the effects of both drugs on mitochondrial function, cellular ATP level and mitochondrial membrane potential were measured. Acetaminophen and diclofenac significantly decreased cellular ATP in rat hepatocytes (61% and 65% of ATP levels in controls, respectively), and in human hepatocytes (28% and 59% of control ATP levels respectively) (Fig 2A and 2B). Both drugs also significantly reduced the fluorescent signals of TMRE/MitoTracker Green to 62% and 72% of controls for rat hepatocytes, and 68% and 44% of controls for human hepatocytes, indicating reduction in mitochondrial membrane potentials (Fig 2C and 2D). We next examined whether activation AMPK by AICAR could prevent these effects. When hepatocytes were treated with acetaminophen or diclofenac in the presence of AICAR, ATP levels and mitochondrial membrane potentials improved to control levels (Fig 2A, 2B and 2D). These results reveal that acetaminophen or diclofenac cause mitochondrial dysfunction in hepatocytes, and that activation of AMPK by AICAR prevents drug-induced mitochondrial dysfunction. Treatment with AICAR alone significantly increased ATP levels and mitochondrial membrane potential in both rat and human hepatocytes (Fig 2A, 2B and 2D).

**Fig 3:**
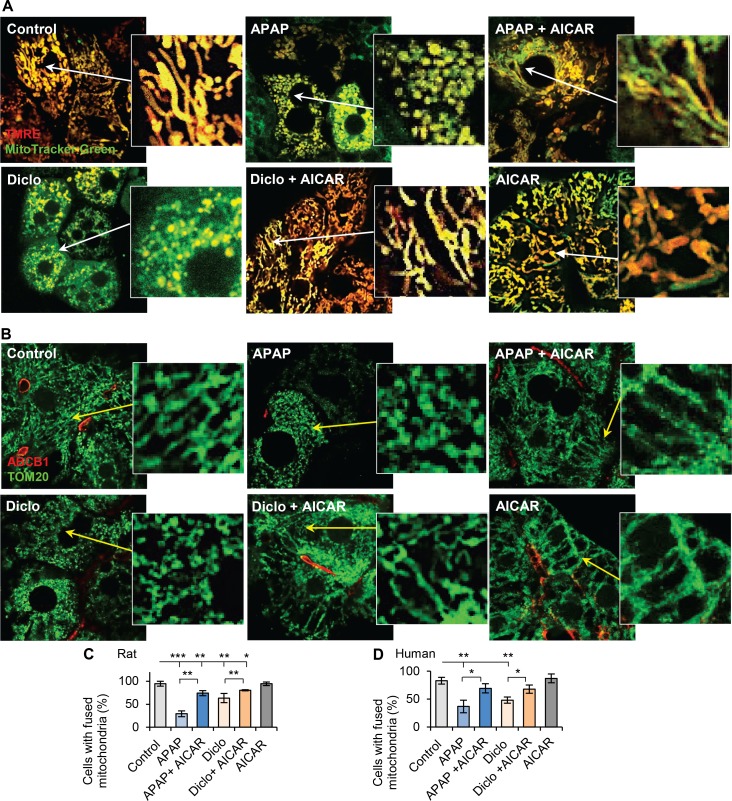
Activation of AMPK prevented drugs-induced mitochondrial fragmentation. (**A**) Rat hepatocytes were treated with acetaminophen (10mM, 24h) or diclofenac (250μM, 24h) with or without AICAR (200μM), and stained with mito-Tracker Green and TMRE (red color). Merged images were taken by confocal microscope (see details in Methods). Both acetaminophen (APAP) and diclofenac (Diclo) caused mitochondrial fragmentation, and AICAR prevented the drug-induced fragmentation. Zoomed areas were indicated. (**B**) Immunofluorescence of Tom20 (green color) and confocal microscope images showed that acetaminophen (25mM, 24h) and diclofenac (1000μM, 24h) caused mitochondrial fragmentation in human hepatocytes, and AICAR prevented the fragmentation. Zoomed areas were indicated. (**C-D**) The numbers of hepatocytes with fused mitochondria were counted and percentages of hepatocytes with fused mitochondria were calculated (see details in Methods). Addition of AICAR to acetaminophen or diclofenac prevented the decrease in percentage of hepatocytes with fused mitochondria. The percentages of rat hepatocytes with fused mitochondria were 94% in Control; 29% in APAP; 74% in APAP + AICAR; 63% in Diclo; 80% in Diclo + AICAR and 94% in AICAR. In human hepatocytes, they were 83% in Control; 37% in APAP; 69% in APAP + AICAR; 48% in Diclo; 68% in Diclo + AICAR and 87% in AICAR (* p<0.05, ** p<0.01 and *** p<0.001).

To gain insights into the preventive effect of AMPK activation on drug-induced mitochondrial dysfunction, mitochondrial fusion/fission morphology was examined using mitochondrial staining in live cells or immunofluorescence of the mitochondrial marker Tom20. Results revealed mitochondrial fragmentation after treatment with acetaminophen or diclofenac in rat hepatocytes (Fig 3A), with only 29% and 63% of hepatocytes having fused mitochondria with the respective drugs, whereas mitochondria remained predominantly fused in control hepatocytes (94%) (Fig 3C). AICAR increased the percentage of hepatocytes with fused mitochondria in the presence of acetaminophen or diclofenac (74% and 80% respectively) (Fig 3A and 3C). Similarly, only 37% and 48% of human hepatocytes treated with acetaminophen or diclofenac contained fused mitochondria, compared to 83% in control human hepatocytes. Addition of AICAR resulted in ~70% human hepatocytes having fused mitochondria (Fig 3B and 3D). These results reveal that hepatotoxic drugs disrupt mitochondrial fusion and that AMPK activation facilitates mitochondrial fusion thereby preventing drug-induced mitochondrial fragmentation.

**Fig 4:**
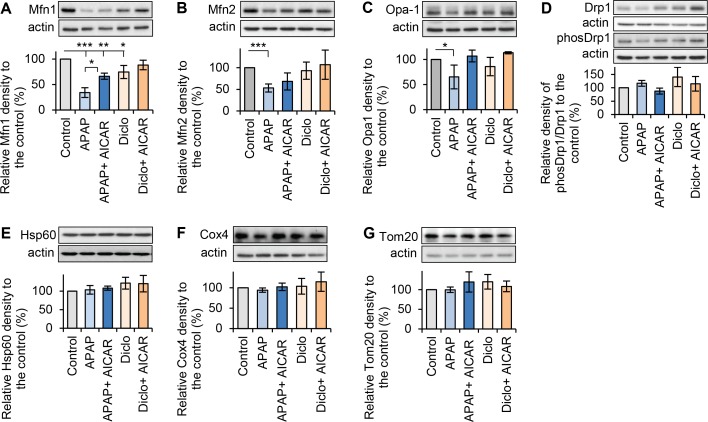
Effects of acetaminophen and diclofenac on expressions of mitochondrial fusion and fission proteins. Total proteins were extracted after rat hepatocytes were treated with acetaminophen (APAP, 10mM, 24h) or diclofenac (Diclo, 250μM, 24h) in the presence or the absence of 200μM AICAR. Western blots were performed to detect protein expressions. Quantitative analyses were obtained by calculating the relative densities compared to respective controls. (**A**) Treatment with acetaminophen or diclofenac significantly decreased Mfn1 expression. Addition of AICAR significantly promoted Mfn1 expression in the presence of both drugs. The relative levels of Mfn1 were 34% in APAP; 66% in APAP + AICAR; 75% in Diclo and 88% in Diclo + AICAR. (**B**) Treatment with acetaminophen, but no diclofenac, decreased Mfn2. AICAR prevented acetaminophen-induced decrease in Mfn2. The relative levels of Mfn2 were 53% in APAP; 68% in APAP + AICAR; 93% in Diclo and 107% in Diclo + AICAR. (**C**) Treatment with acetaminophen or diclofenac reduced Opa1expression. AICAR prevented drug-induced decrease in Opa1. The relative levels of Opa1 were 65% in APAP; 107% in APAP + AICAR; 86% in Diclo and 113% in Diclo + AICAR. (**D**) Treatment with acetaminophen or diclofenac in the presence or the absence of AICAR did not have significant effects on activity of Drp1, measured by the ratio of phos-Drp1 (616) to total Drp1, and the expressions of mitochondrial structure proteins, such as (**E**) Hsp60, (**F**) Cox4 and (**G**) Tom20 (* p<0.05, ** p<0.01 and *** p<0.001).

To elucidate how hepatotoxic drugs cause mitochondrial fragmentation and how AMPK activation maintains mitochondrial fusion, the expressions of mitochondrial fusion/fission proteins were examined in rat hepatocytes. Western blot analyses showed that acetaminophen significantly down-regulated fusion proteins Mfn1, Mfn2 and Opa1 to 34%, 53% and 65% of expression levels in the respective controls (Fig 4A, 4B and 4C). Diclofenac also significantly decreased Mfn1 to 75% of the level in controls, but did not decrease expressions of Mfn2 and Opa1 (Fig 4A, 4B and 4C). Those results suggested that both drugs down-regulated the fusion machinery. The mechanism was not studied further. Both hepatotoxic drugs may down-regulate mitochondrial fusion proteins by transcriptional and/or post transcriptional (e.g. altered stability) mechanisms. In the presence of acetaminophen, addition of AICAR significantly increased Mfn1 to 66% of that in control (Fig 4A). AICAR also prevented acetaminophen-induced decreases in both Mfn2 and Opa1, and increased their levels to 68% and 107% of that in respective controls (Fig 4B and 4C). Moreover, AICAR prevented the decrease in Mfn1 in the presence of diclofenac, and increased Mfn1 expression to 88% of that in control (Fig 4A). However, AICAR did not fully restore Mfn1 and Mfn2 expressions in hepatocytes treated with acetaminophen (Fig 4A and 4B). Nevertheless, those results indicated that addition of AICAR prevented drug-induced down-regulation of mitochondrial fusion proteins. Neither acetaminophen nor diclofenac affected activity of the fission protein Drp1 as measured by the ratio of phos-Drp1 (Ser616) to total Drp1 (Fig 4D), or the expressions of mitochondrial structural proteins Hsp60, Cox4 and Tom20 (Fig 4E, 4F and 4G). Taken together, hepatotoxic drugs induced mitochondrial fragmentation via down-regulation of mitochondrial fusion proteins. AMPK activation helps to maintain mitochondrial fusion possibly by enhancing fusion machinery, thereby promoting mitochondrial quality and function under stress conditions generated by the drugs.

### AMPK activator AICAR promotes mitochondrial fusion by upregulating fusion proteins Mfn1 and Opa1

**Fig 5:**
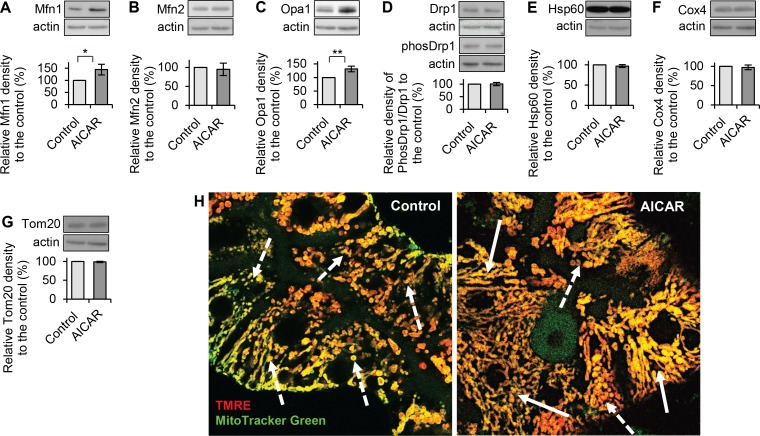
Effect of AMPK activator AICAR on expressions of mitochondrial fusion and fission proteins. Total proteins were extracted after hepatocytes were treated with AICAR (200μM, 24h). Western blots were performed to detect protein expressions. Quantitative analyses were conducted by calculating the relative densities compared to the respective controls. (**A**) AICAR significantly increased Mfn1 level to 145% of that in the control. (**B**) AICAR did not affect Mfn2 expression (95% of that in the control). (**C**) AICAR significantly increased Opa1 level to 132% of that in the control. (**D**) AICAR did not affect activity of fission protein Drp1 measured by ratio phos-Drp1 (ser 616) to total Drp1 (100% of that in the control). (**E-G**) AICAR did not affect expressions of mitochondrial structure proteins Hsp60, Cox4 and Tom20 (* p<0.05 and ** p<0.01). (**H**) Representative images of mitochondrial staining of TMRE (red) and MitoTracker Green showed that more interconnected mitochondrial network were presented in hepatocytes treated with AICAR (indicated by the white-solid arrows). In the control hepatocytes, there were less interconnected mitochondrial network, and mainly contained a mixture of fused and fragmented mitochondria (indicated by the white-dots arrows).

To investigate how AICAR promotes mitochondrial fusion, hepatocytes were treated with or without AICAR (200μM, 24hrs). Western blot was performed to examine the expressions of mitochondrial fusion and fission proteins. AICAR significantly increased the expressions of fusion proteins Mfn1 and Opa1 to 145% and 132% of that in respective controls, but did not affect Mfn2 expression (Fig 5A, 5B and 5C). AICAR did not show any effect on activity of fission protein Drp1 (measured by ratio of phos-Drp1 (616) to total Drp1) (Fig 5D) and the expressions of mitochondrial structure proteins Hsp60, Cox4 and Tom20 (Fig 5E, 5F and 5G). These results suggest that AICAR enhances mitochondrial fusion through promoting the fusion machinery (i.e. Mfn1 and Opa1). Images from confocal microscope indicated that more hepatocytes had interconnected mitochondrial network when they were treated with AICAR, whereas many control hepatocytes had less interconnected mitochondrial network, and mainly contained a mixture of fused and fragmented mitochondria (Fig 5H).

**Fig 6:**
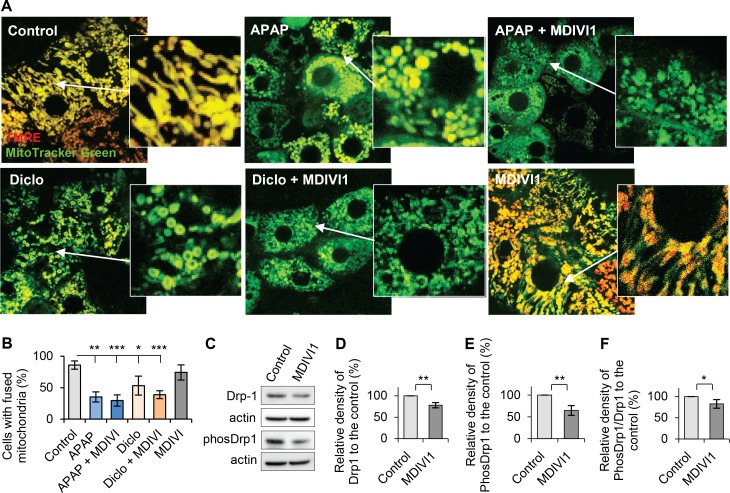
Inhibition of mitochondrial fission by Drp1 inhibitor MDIVI1 did not prevent drug-induced mitochondrial fragmentation. Hepatocytes were treated with acetaminophen (APAP) or diclofenac (Diclo) with or without Drp1 inhibitor, MDIVI1 (25μM). (**A**) Mito-staining and confocal microscope images showed that MDIVI1 did not prevent acetaminophen- and diclofenac-induced mitochondrial fragmentation in rat hepatocytes. (**B**) Percentages of hepatocytes with fused mitochondria were calculated and analysed (see details in Methods). MDIVI1 did not have effect on the percentages of hepatocytes with fused mitochondria in the presence of acetaminophen or diclofenac. The percentages of cells with fused mitochondria were 86% in Control; 35% in APAP; 30% in APAP + MDIVI1; 53% in Diclo; 39% in Diclo + MDIVI1 and 75% in MDIVI1. (**C**) Hepatocytes were treated with or without MDIVI1 (25μM, 24hr). Western blot were performed to detect Drp1 and phos-Drp1(616). Quantitative analyses were obtained by calculating the relative densities compared to respective controls. (**D**) Quantitative analysis for expression of Drp1. (**E**) Quantitative analysis for expression of phos-Drp1(616). (**F**) Quantitative analysis for activity of Drp1 (ratio of phos-Drp1/Drp1) (* p<0.05, ** p<0.01 and *** p<0.001).

To examine the role of mitochondrial fission in drug-induced mitochondrial fragmentation, hepatocytes were treated with a selective cell-permeable inhibitor of Drp1, MDIVI1 (3-(2,4-Dichloro-5-methoxyphenyl)-2,3-dihydro-2-thioxo-4(1H)-quinazolinone) [43]. Morphological examination and quantitative analyses showed that the percentages of cell with fused mitochondria were similar to that in controls when hepatocytes were treated with acetaminophen or diclofenac and MDIVI1 (25μΜ). This suggested that MDIVI1 did not prevent acetaminophen- or diclofenac-induced mitochondrial fragmentation in rat hepatocytes (Fig 6A and 6B). Western blot results showed that MDIVI1 decreased expression levels of both Drp1 and phosDrp1(616) (Fig 6C, 6D and 6E) as well as the activation of Drp1(Fig 6F), indicating its inhibition of Drp1. These results confirm that acetaminophen or diclofenac-induced mitochondrial fragmentation is independent from Drp1. Mitochondria in hepatocytes treated with MDIVI1 alone did not show increased fusion (Fig 6B). Decreased Drp1 alone may be insufficient to promote mitochondrial fusion. It is also possible that the percentage of cells with fused mitochondria was already high in the control cells (86%).

### The autophagic activity of AMPK has differential effect on prevention of drug-induced hepatocellular injury

**Fig 7:**
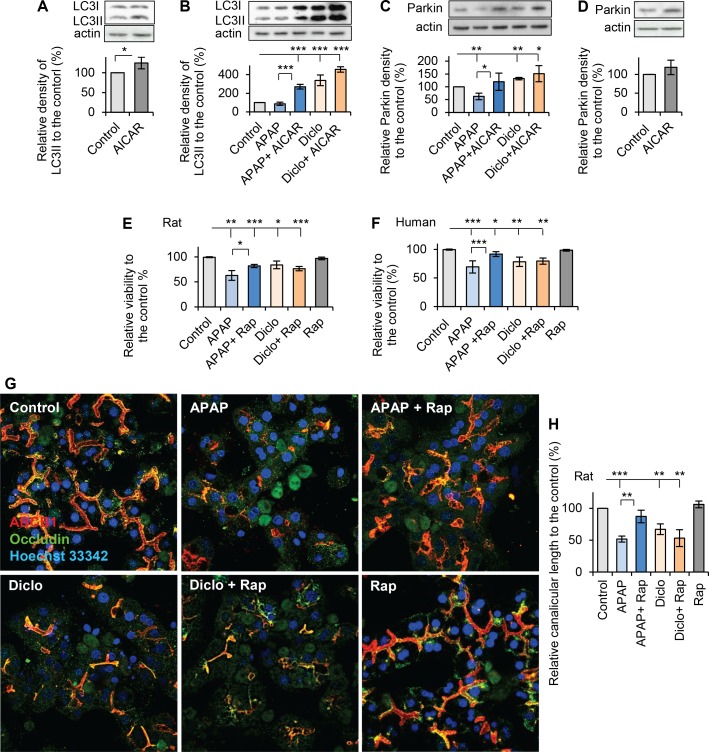
Activation of autophagy had differentially effects on prevention of drug-induced hepatocellular injury. Rat hepatocytes were treated with acetaminophen (APAP, 10mM, 24h) or diclofenac (Diclo, 250μM, 24h) with or without AICAR (200μM). Western blots for autophagic marker LC3II and mitophagy marker Parkin were performed. Quantitative analyses were conducted by calculating the relative densities compared to the respective controls. (**A**) Treatment with 200μM AICIAR alone in hepatocytes significantly increased LC3II expression to 125% of that in the control, confirming that activation of AMPK promotes autophagy activity. (**B**) Acetaminophen only mildly decreased LC3II expression. AICAR significantly increased LC3II level in the presence of acetaminophen. Diclofenac significantly increased LC3II levels, and addition of AICAR to diclofenac did not elevate the LC3II significantly. The relative levels of LC3II were 88% in APAP; 270% in APAP + AICAR; 338% in Diclo and 456% in Diclo + AICAR. (**C**) Acetaminophen significantly decreased Parkin expression to 62% of that in the control, and addition of AICAR prevented the decrease. Diclofenac significantly increased Parkin level to 132% of that in the control, and addition of AICAR to diclofenac did not elevate the Parkin significantly. The relative levels of Parkin were 62% in APAP; 120% in APAP + AICAR; 132% in Diclo and 151% in Diclo + AICAR. (**D**) Treatment with 200μM AICIAR alone in hepatocytes mildly, but not significantly, increased Parkin expression (119% of that in the control). (**E**) Autophagy activator rapamycin (2μM, 24hr) significantly improved viability in acetaminophen-treated rat hepatocytes, but not in diclofenac-treated rat hepatocytes. Treatment of rapamycin (Rap) alone did not significantly change viability. The relative viabilities were 63% in APAP; 82% in APAP + Rap; 84% in Diclo; 76% in Diclo + Rap and 97% in Rap. (**F**) Human hepatocytes were also treated with acetaminophen (25mM, 24h) or diclofenac (1000μM, 24h) in the presence or absence of rapamycin (2μM). Similarly, rapamycin prevented acetaminophen-induced, but not diclofenac-induced, decrease in viability. Treatment of rapamycin alone did not significantly change viability. The relative viabilities were 69% in APAP; 92% in APAP + Rap; 78% in Diclo; 79% in Diclo + Rap and 98% in Rap. (**G**) Representative images from confocal microscope showed that rapamycin prevented acetaminophen-induced, but not diclofenac-induced, depolarization. (**H**) Quantitative analyses of polarization by measuring canalicular lengths showed the rapamycin prevented acetaminophen-induced, but not diclofenac-induced, decrease in canalicular length in hepatocytes. The relative canalicular lengths were 52% in APAP; 87% in APAP + Rap; 67% in Diclo; 53% in Diclo + Rap and 106% in Rap. (* p<0.05, ** p<0.01 and *** p<0.001).

Autophagy plays an important role in response to cellular stress and eliminating damaged mitochondria, a process known as mitophagy [44, 45]. Activation of AMPK induces autophagic activity [46], and Western blot of autophagy marker LC3II (microtubule-associated protein 1A/1B-light chain 3-II) showed that LC3II was significantly increased to 125% of that in controls after addition of AICAR (200μM, 24hrs) (Fig 7A), confirming that activation of AMPK increased autophagy. To determine whether the preventive effects of AMPK against drug-induced hepatocyte injury also include activation of autophagy, expression of LC3II was analysed by Western blot in hepatocytes exposed to acetaminophen or diclofenac with and without AICAR. Hepatocytes exposed to acetaminophen only slightly, but not significantly, reduced LC3II level to 88% of that in the control whereas hepatocytes incubated with diclofenac significantly increased LC3II level to 338% of that in controls. In acetaminophen-treated hepatocytes, addition of AICAR significantly increased LC3II levels from 88% to 270% of that in controls. In diclofenac-treated hepatocytes, AICAR did not significantly increased LC311 which was already elevated by diclofenac (Fig 7B). Notably, the stimulatory effect of AICAR regarding autophagy was much stronger in presence of APAP than in cells treated AICAR alone. It is possible that other autophagy pathways are activated by synergy between AICAR and APAP. Moreover, both drugs also showed different effects on mitophagy as measured by expression of mitophagy marker Parkin [47]. Acetaminophen significantly decreased Parkin level to 62% of that in controls; however, diclofenac increased Parkin level to 132% of that in controls (Fig 7C). Addition of AICAR to acetaminophen significantly increased Parkin level to 120% of that in controls; however, addition of AICAR to diclofenac only slightly, but not significantly, increased Parkin levels (Fig 7C). Taken together, acetaminophen and diclofenac have different effects on autophagy/mitophagy. Acetaminophen slightly reduced autophagy, and caused significant decrease in mitophagy. However, diclofenac increased both activities of autophagy and mitophagy. AICAR significantly promoted autophagy/mitophagy in hepatocytes treated with acetaminophen, but it only slightly increased autophagy/mitophagy in hepatocytes treated with diclofenac. These results indicated that the autophagic effect of AMPK differs in preventing acetaminophen-induced versus diclofenac-induced hepatocellular injury. In addition, treatment with AICAR alone only mildly, but not significantly, increased Parkin level in hepatocytes (Fig 7D).

To confirm these different effects of autophagy, autophagy activator, rapamycin, was introduced to hepatocytes in the presence or absence of acetaminophen or diclofenac. Rapamycin prevented acetaminophen-induced cell damage; and significantly improved viabilities from 63% to 82% in rat hepatocytes, and from 69% to 92% in human hepatocytes (Fig 7E and 7F). In contrast, rapamycin did not improve viability of rat or human hepatocytes treated with diclofenac (Fig 7E and 7F). Moreover, rapamycin also prevented acetaminophen-induced, but not diclofenac-induced, depolarization (Fig 7G); and addition of rapamycin increased canalicular length in acetaminophen treated hepatocytes from 52% to 87% of that in the control, but rapamycin did not affect canalicular length in diclofenac-treated hepatocytes (Fig 7H). Those results confirm that activation of autophagy, either by rapamycin or AMPK activator AICAR, had different effects on prevention of acetaminophen-induced and diclofenac-induced hepatocyte injury and depolarization. This is possibly associated with different effects of those drugs on autophagy/mitophagy.

**Fig 8:**
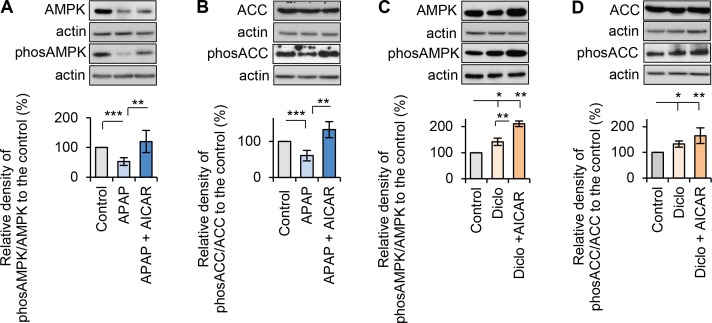
Acetaminophen and diclofenac had differential effects on AMPK activation. Rat hepatocytes were treated with acetaminophen (APAP, 10mM, 24h) or diclofenac (Diclo, 250μM, 24h) in the presence or the absence of AICAR (200μM). Western blots of AMPK, phos-AMPK (Thr172), ACC and phos-ACC (Ser79) were performed. Ratios of phosphorylated protein to the total protein were used to measure the activation of AMPK or ACC, and percentages of the relative ratios were calculated by comparing to the respective controls. (**A**) Acetaminophen significantly deactivated AMPK, and addition of AICAR prevented the deactivation. The relative activities of AMPK were 53% in APAP and 120% in APAP + AICAR. (**B**) Acetaminophen also significantly reduced phos-ACC (ser79) levels confirming the deactivation of AMPK. AICAR prevented acetaminophen-induced decrease in phos-ACC. The relative levels of ACC were 61% in APAP and 133% in APAP + AICAR. (**C**) Diclofenac activated AMPK, and addition of AICAR significantly promoted the activation of AMPK. The relative activities of AMPK were 142% in Diclo and 211% in Diclo + AICAR. (**D**) Diclofenac also significantly increased phos-ACC (ser 79) levels confirming the activation of AMPK. Addition of AICAR increased, but not significantly, the level of phos-ACC in the presence of diclofenac. The relative levels of ACC were 132% in Diclo and 165% in Diclo + AICAR (* p<0.05, ** p<0.01 and *** p<0.001).

To gain insight why acetaminophen and diclofenac have different effects on autophagy/mitophagy, their effects on activation of AMPK were examined. Acetaminophen significantly deactivated AMPK to 53% of that in controls (Fig 8A). Acetaminophen also decreased AMPK substrate phos-ACC level to 61% of that in controls (Fig 8B). AICAR prevented acetaminophen-induced deactivation of AMPK and the decrease in phos-ACC, and significantly promoted AMPK activation and phos-ACC to 120% and 133% of that in controls, respectively (Fig 8A and 8B). In contrast, diclofenac treatment significantly activated AMPK (~142% of the level in controls) and increased phos-ACC (132% of the level in controls) (Fig 8C and 8D). Addition of AICAR to diclofenac promoted AMPK activity significantly (211% of that in controls), and also increased, but not significantly, the level of phos-ACC level (165% of that in controls) (Fig 8C and 8D). These results suggest that inhibition of AMPK activation by acetaminophen is possibly associated with decreased autophagy/mitophagy and hepatocyte damage, whereas diclofenac-induced hepatocellular injury may occur independently from AMPK activation. It is unclear why the drugs have different effects on AMPK activation, and further studies are needed to explore this.

### AMPK activation reverses drug-induced hepatocyte injury

**Fig 9:**
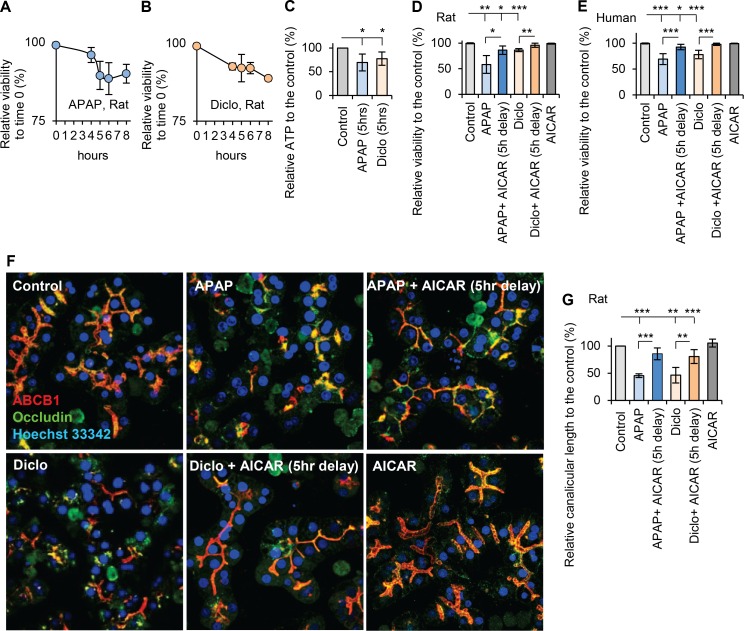
AMPK activation reversed drug-induced hepatocellular injury. Rat hepatocytes were treated with 10mM acetaminophen (APAP) or 250μM diclofenac (Diclo) for a period of 8 hours. Viabilities were measured every hour and time-courses of viability were analysed for (**A**) acetaminophen, and (**B**) diclofenac. (**C**) ATP levels were measured after rat hepatocytes were treated with 10mM acetaminophen or 250μM diclofenac for 5 hours. Both drugs significantly decreased ATP levels to 70% and 77% of that in the control, respectively. (**D-E**) To investigate the reversal effect of AICAR, hepatocytes were first treated with acetaminophen (10mM for rat hepatocytes; 25mM for human hepatocytes) or diclofenac (250μM for rat hepatocytes; 1000μM for human hepatocytes) for 5 hours, hepatocytes were further incubated with or without 200μM AICAR for additional 18 hours. Viabilities were examined. After rat or human hepatocytes were incubated with acetaminophen or diclofenac for 5hr, the viabilities were significantly lower than that in respective controls. The administration of AICAR significantly improved viabilities. The relative viabilities in rat hepatocytes were 58% in APAP; 86% in APAP + AICAR; 86% in Diclo; 96% in Diclo +AICAR and 99% in AICAR. In human hepatocytes, the relative viabilities were 69% in APAP; 93% in APAP + AICAR; 78% in Diclo; 98% in Diclo +AICAR and 100% in AICAR. (**F**) Immunofluorescence and confocal microscope were used to examine polarized morphology with the same treatments stated in D-E. Representative images showed that addition of AICAR at 5hr post exposure of acetaminophen or diclofenac reversed drug-induced depolarization. (**G**) Quantitative analyses of polarization by measuring canalicular lengths showed that AICAR reversed the acetaminophen- or diclofenac-induced decrease in canalicular lengths. The relative canalicular lengths were 45% in APAP; 86% in APAP + AICAR; 47% in Diclo; 81% in Diclo +AICAR and 105% in AICAR (* p<0.05, ** p<0.01 and *** p<0.001).

Since there is no effective treatment for DILI once liver damage occurs, we studied whether AMPK activation can reverse drug-induced hepatocyte injury. Viabilities were measured every hour and time-courses of viability were obtained after hepatocytes were treated with acetaminophen or diclofenac for a period of 8 hours. After 5 hours treatment of acetaminophen or diclofenac, viabilities decreased to 89% and 91% respectively (Fig 9A and 9B), and cellular ATP were reduced to 70% and 77% of that in controls, respectively (Fig 9C). AICAR (200μM) was added to rat or human hepatocytes 5 hours after exposure to acetaminophen or diclofenac and continued the incubation for an additional 18hrs. Addition of AICAR significantly improved viabilities in both rat and human hepatocytes. AICAR increased viability from 58% to 86% in acetaminophen-treated rat hepatocytes and from 86% to 96% in diclofenac-treated rat hepatocytes. In human hepatocytes, AICAR increased the viabilities from 69% to 93% and from 78% to 98% for acetaminophen and diclofenac treatments, respectively (Fig 9D and 9E). Addition of AICAR also maintained normal polarized morphology in acetaminophen- or diclofenac-treated hepatocytes (Fig 9F), and increased the canalicular length from 45% to 86% of that in controls in acetaminophen-treated hepatocytes, and from 47% to 81% of that in controls in diclofenac-treated hepatocytes (Fig 9G). However, AICAR did not reverse cellular injury when added later than 5hrs post treatment with acetaminophen or diclofenac. These results suggest that AMPK activation can reverse drug-induced hepatocellular injury at early stages before cellular stress leads to irreversible damage.

## Discussion

Although mitochondria play a central role in DILI [1, 37, 48] and both acetaminophen and diclofenac cause mitochondrial injury [39–41], the mechanism responsible for drug-induced mitochondrial damage are still unclear and strategies for preventing DILI through promoting mitochondrial function have not been investigated. Using collagen sandwich cultures of rat and human hepatocytes, we demonstrated that two pharmacologically different hepatotoxic drugs, acetaminophen and diclofenac, impaired mitochondrial fusion resulting in mitochondrial fragmentation and dysfunction which result in hepatocyte damage. Activation of AMPK by AICAR maintained normal mitochondrial morphology and function, and prevented drug-induced mitochondrial and hepatocellular injury. Moreover, activation of AMPK reversed the early stage of drug-induced mitochondrial and hepatocellular injury.

Mitochondrial fission and fusion, which are regulated by specific fusion and fission proteins, are critical for mitochondrial function and quality control [12, 49]. We found that mitochondrial fragmentation is associated with drug-induced hepatocyte injury and is related to inhibition of the mitochondrial fusion machinery. Specifically, acetaminophen and diclofenac down-regulated the mito-fusion proteins Mfn1, Mfn2 and Opa1, but did not affect the activity of fission protein Drp1. Inhibition of fission by Drp1 inhibitor MDIVI1 did not prevent fragmentation, confirming acetaminophen- and diclofenac-induced mitochondrial fragmentation are independent from Drp1. Reduction of membrane potential can also lead to fragmentation of mitochondria in a Drp1 independent manner [50].

Stress-induced mitochondrial fragmentation enhances mitophagy and removal of damaged mitochondria [51]. Down-regulation of Mfn1, Mfn2 and Opa1 prevented subsequent mitochondrial fusion which is required for optimal mitochondrial function. AMPK activation increased expression of fusion proteins (i.e. Mfn1and Opa1) thereby promoting mitochondrial fusion in the presence of acetaminophen or diclofenac. It is likely that AMPK activation helps override mitochondrial toxicity of the drugs by enhancing fusion of healthy ‘daughter’ mitochondria or newly synthesised mitochondria after mitochondria become fragmented and are processed for mitophagy. In addition, by initiating mitochondria biogenesis [19, 52], AMPK activation may aid in stabilizing mitochondrial function and cell survival. It is unclear whether mitochondrial fusion protein(s), such as Mfn1 and Opa1, are direct downstream targets of AMPK or are indirectly affected by AMPK activation. Computer modelling using protein sequences does not indicate potential interactions between AMPK and Mfn1 or Opa1; and the consensus AMPK motif is not found in Mfn1 and Opa1. Because mitochondrial quality control is a multi-event process, promotion of fusion alone may not be sufficient to overcome mitochondrial damage.

A recent study reported that AMPK activation (1hr, 2mM AICAR) mediated mitochondria fragmentation in mouse embryonic fibroblasts and hepatocytes under induced stress [53]. In contrast, our results indicate that AMPK activation by AICAR (24hr, 200μM) is associated with mitochondrial fusion even in the presence of toxic drugs. Furthermore, AMPK activation by AICAR (500μM, 24hr or 1mM, 24hr or 2mM, 1hr) did not cause mitochondrial fragmentation in collagen sandwich cultures of rat hepatocytes. Another study also showed that activation of AMPK by AICAR (24hr, 750μM) did not lead to mitochondrial fragmentation in various cancers cells [54]. A recent report suggested that AICAR (24hr, 2mM) prevented cisplatin-induced mitochondrial fragmentation in human renal proximal tubule epithelial cells and in vivo [55]. The different effects of AICAR on mitochondrial fusion/fission may be due to the duration of treatment and the stress status of the cells. It is possible that activation of AMPK by AICAR has dual roles in regulating mitochondrial fusion/fission dynamics. When mitochondria are damaged and beyond repair and cells are under extreme stress, AMPK-mediated mitochondrial fragmentation may become a dominant effect which enables rapid removal of damaged mitochondria and prevents cell injury. In the longer term, after elimination of damaged mitochondria and cell stress is reduced, AMPK-mediated mitochondrial fusion may become more effective in restoring mitochondrial function. Therefore, the possible dual effects of AMPK on mitochondrial fusion and fission could promote mitochondrial function as well as hepatocyte viability.

AMPK also promotes mitochondrial function and quality through induction of autophagy [46]. AICAR had different effects on autophagy/mitophagy in hepatocytes treated with acetaminophen or diclofenac. In acetaminophen-treated cells, AMPK activation significantly increased autophagy/mitophagy, but there was no significant effect in diclofenac-treated cells in which autophagy/mitophagy levels were already markedly increased. Consistent with this, when we directly activated autophagy using rapamycin, only acetaminophen-induced hepatocytes injury was reduced, as reported in a previous study [56]. The difference in autophagy/mitophagy activity may be associated with different effects of the two hepatotoxic drugs on AMPK activation. Acetaminophen down-regulates and deactivates AMPK which inhibits AMPK downstream effects (e.g. autophagy, mitochondrial biogenesis and fusion), and leads to cellular stress and damage. Thus activation of autophagy alone by AMPK may be a critical target in treatment of acetaminophen-induced hepatocellular injury. However, a different scenario occurs in diclofenac case. Diclofenac increases AMPK and activates autophagy, which may limit further promotion of autophagy. Other hepatotoxic drugs have different effects on mitophagy. Anti-tuberculosis drugs (e.g. rifampicin, isoniazid) induce mitophagy in HepG2 cells [57], and clinical concentrations of efavirenz promote mitophagy [58]. In contrast, perfluorooctane sulfonate impaired mitophagy in HepG2 cells [59], and amitriptyline impaired mitophagy in human hepatocytes [60]. It is possible that the different stress/damage levels cause by hepatotoxic drugs lead to opposite effects on mitophagy [58].

Notably, AMPK activation reverses both acetaminophen and diclofenac-induced hepatocyte injury, however, the effect of AMPK was associated with the severity and duration of mitochondrial and hepatocellular damage. AMPK had a more profound reversal effect for hepatocytes treated with a lower dose of drugs or at an earlier stage (≤ 5hr) of toxicity in which mitochondrial functions were maintained or restored by quality control [61]. Temporary loss of mitochondrial membrane potential may be reversible whereas severe mitochondrial membrane damage may be an irreversible event resulting in apoptosis or necrosis. Thus, only hepatocytes that experience minor and temporary mitochondrial dysfunction/injury are rescued by AMPK activation. Nevertheless, besides its reversal effect, AMPK also can prevent potential damage to healthy mitochondria thereby promoting cell survival.

**Fig 10:**
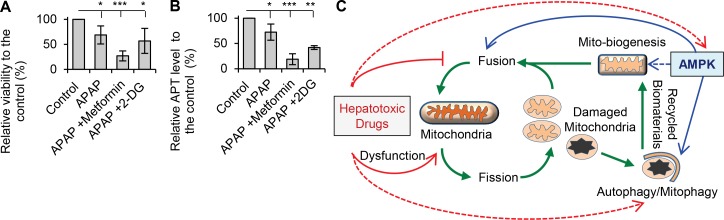
(**A**) Rat hepatocytes were treated with acetaminophen (APAP, 10mM, 24hr) alone or in addition of metformin (100μM) or 2-DG (2-deoxy-D-glucose) (200mM). Metformin and 2-DG were unable to prevent acetaminophen-induced decrease in viability. The relative viabilities were 69% in APAP; 27% in APAP + Metformin and 57% in APAP + 2-DG. (**B**) Metformin and 2-DG were unable to prevent acetaminophen-induced decrease in cellular ATP. The relative ATP levels were 72% in APAP; 19% in APAP + Metformin and 42% in APAP + 2-DG (* p<0.05, ** p<0.01 and *** p<0.001). (**C**) **Model of AMPK effects on drug-induced mitochondrial and hepatocyte injury**. **Green** lines indicate mitochondrial biogenesis, fusion/fission dynamic and autophagic degradation (mitophagy). Mitochondria undergo fusion/fission, the fused mitochondria have higher oxidative capacity. Mitochondria process fragmentation when they are damaged so as to separate the damaged mitochondria. The damaged mitochondria are sent to autophagy for degradation (mitophagy) and the undamaged ‘daughter’ mitochondria can be fused again. The biomaterial from mitophagy will be used for synthesis new mitochondria. **Red** lines indicate the effects of hepatotoxic drugs on mitochondria and AMPK. Acetaminophen and diclofenac cause mitochondrial dysfunction and damage. Both drugs decrease expression of mitofusion proteins resulting in mitochondrial fragmentation and decreased function. Hepatotoxic drugs may also inhibit AMPK activation and affect its downstream (i.e. mito-biogenesis and autophagy). Hepatotoxic drugs can also directly inhibit autophagy/mitophagy (red dots line). **Blue** lines indicate the regulatory effects of AMPK on mitochondrial function and quality control, including induction of autophagy which is responsible for removal of damaged mitochondria; increases in mito-biogenesis which is essential for maintain the quantity of mitochondria during cellular stress. The current study reveals that activation of AMPK increases mito-fusion protein level and promotes fusion to maximise the oxidative capacity during stress.

Several pharmacological reagents, including metformin and 2-DG (2-deoxy-D-glucose), directly or indirectly activate AMPK in hepatocytes. However, although metformin and 2-DG lead to AMPK activation [28], neither prevented acetaminophen-induced hepatocyte injury. Compared to treatment with acetaminophen alone, addition of metformin or 2-DG further decreased viability (Fig 10A). Both metformin and 2-DG also accelerated ATP depletion in the presence of acetaminophen (Fig 10B). Metformin and 2-DG may contribute to mitochondrial stress in acetaminophen-treated cells despite activating AMPK. Metformin inhibits mitochondrial respiration [62] and causes mitochondrial dysfunction [63], whereas 2-DG inhibits glycolysis and further reduces cellular ATP levels. In contrast to metformin and 2-DG, AICAR is an AMP analog, which increases the cellular ratio of AMP/ATP and directly activates AMPK. Thus, it may be that only direct activators of AMPK, such as AICAR, will be successful agents for treatment of drug-induced hepatocellular injury. AICAR also has other targets independently from AMPK activation, such as JNK (c-jun N-terminal kinase) and Jak/STAT (Janus kinase) [64, 65]. Activation of JNK and translocation of phos-JNK to the mitochondria are important in acetaminophen-mediated DILI, and inhibition of JNK prevents acetaminophen-induced DILI [66–68]. AICAR also inhibited JNK activity in HepG2 [69] and retinal cells [70]. Thus, the preventive effect of AICAR on drug-induced hepatotoxicity may also be linked to inhibition of JNK. However, other studies showed that AICAR activates JNK and induces apoptosis in insulinoma [71] and prostate cancer cells [72]. Therefore, more investigations are needed to determine whether JNK is involved in the preventive effect of AICAR on drug-induced liver injury.

In summary, our study reveals that impairment of mitochondrial fusion is an important mechanism in drug-induced mitochondrial and hepatocyte injury. Inhibition of autophagy/mitophagy is also a mechanism for hepatotoxicity of drugs, such as acetaminophen. Activation of AMPK prevents and reverses mitochondrial and hepatocellular injury through several mechanisms including promotion of mitochondrial fusion and enhanced autophagy-mediated removal of damaged mitochondria [73, 74], increased mitochondrial biogenesis [20] and maintenance of hepatocellular polarization (Fig 10C). These observations suggest that drugs that activate AMPK may help reverse drug-induced mitochondrial injury and constitute a viable approach for treating DILI. It remains to be determined whether AMPK activation can be similarly effective for hepatotoxicity caused by other drugs than those studied. Additional studies are needed to determine whether AMPK activation also enhances the activity of drug efflux transporters to minimize hepatocellular accumulation of toxic drugs/metabolites in addition to its effects on mitochondria.
